# Characterizing Soil Dissolved Organic Matter Across a Permafrost Thaw Gradient (Continuous to Isolated Patches) in Northeastern China

**DOI:** 10.1002/ece3.71667

**Published:** 2025-07-03

**Authors:** Siyuan Zou, Xiaodong Wu, Jiawei Zhang, Nannan Zhang, Xiangwen Wu, Shuying Zang

**Affiliations:** ^1^ Heilongjiang Province Key Laboratory of Geographical Environment Monitoring and Spatial Information Service in Cold Regions Harbin Normal University Harbin China; ^2^ Heilongjiang Province Collaborative Innovation Center of Cold Region Ecological Safety Harbin China; ^3^ Cryosphere Research Station on the Qinghai‐Tibet Plateau, State Key Laboratory of Cryospheric Science, Northwest Institute of Eco‐Environment and Resources Chinese Academy of Sciences Lanzhou China

**Keywords:** dissolved organic matter, fluorescence, parallel factor analysis, peatland, permafrost gradient

## Abstract

Permafrost zones are currently experiencing rapid warming, and dissolved organic matter (DOM) is a potentially important pathway for carbon release from permafrost after thawing. In this study, based on the UV–Vis spectral data and parallel factor analysis of fluorescence excitation‐emission matrix (EEM) spectrophotometry, we investigated the source and composition of DOM at different thawing stages in the permafrost zone of Northeast China. The results indicate that there are significant differences in the content of soil dissolved organic carbon (DOC) among different types of permafrost zones (continuous permafrost: 143–347 mg/kg, discontinuous permafrost 172–462 mg/kg, isolated patches permafrost 195–610 mg/kg), and the permafrost types had a significant effect on soil DOC (*p* < 0.05). Five fluorescent components were identified from all samples, including four humic acid‐like substances (C1, C2, C3, C4) and one tryptophan‐like substance (C5). The proportion of C5 in the deep layer of the sporadic patch permafrost area (55.39%) is about 49% higher than that in the continuous permafrost area (37.25%). With increasing soil depth, the characteristics of DOM transition from high DOC value, high aromaticity, high molecular weight, and plant origin to low DOC value, low aromaticity, low molecular weight, and microbial origin. The results of soil DOM component analysis indicate that the content of C5 significantly increases (*p* < 0.01) in the deeper layers of isolated patch permafrost. With the change of permafrost types, the risk of DOM decomposition may increase. These findings contribute to a deeper understanding of the dynamics and stability of DOM in permafrost under environmental warming, as well as its biogeochemical impacts in natural environments.

## Graphical abstract

The DOC content under different permafrost zones gradually decreases from top to bottom, and increases from the continuous permafrost zone to the island patches permafrost zone. The humic acid‐like substances in DOM in different permafrost zones decrease, and the tryptophan‐like substance increase with deeper soil depth. DOM under different permafrost gradients transforms from high quality and high aromaticity to low quality and low aromaticity as the soil depth increases.

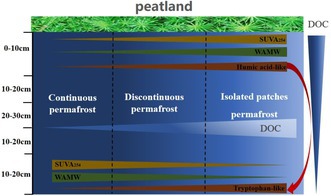

## 1 Introduction

Permafrost affected soils store an estimated 1000 ± 150 Pg organic carbon at 0–3 m depth (Hugelius et al. 2014) Under a warming climate, permafrost degradation has been recorded at the global scale (Biskaborn et al. 2019). The most serious future scenario predicts 14% reduction in the area of near‐surface permafrost by the end of the century (Peng et al. 2023). When permafrost thaws, the active layer may deepen or the frozen layer may disappear completely (Chen et al. 2022). As permafrost degrades over the years, frozen organic matter is broken down and released into the atmosphere as greenhouse gases (Ruggiero et al. 2023), with estimates of permafrost carbon release ranging from 28 to 113 Pg by 2100 under the RCP8.5 scenario (Koven et al. 2015). In particular, under long‐term cold and wet conditions, northern peatlands have accumulated twice the carbon content of the atmosphere, accounting for approximately 1/3 of global carbon reserves (Tarnocai et al. 2009; Yu et al. 2010). It is therefore critical to understand the strength of potential feedbacks on climate change from permafrost melting in peatlands.

Dissolved organic matter (DOM) is a widespread and vital component of both natural soil and aquatic ecosystems (Kaiser et al. 2004). In its nature, DOM is active and mobile, serving as a crucial substrate for microorganisms and playing a key role in the overall net carbon balance within permafrost ecosystems (McGuire et al. 2010). Despite making up only < 0.5%–1% of soil organic carbon (SOC), soil DOM is the most reactive and mobile organic matter component in soil, and it has a significant impact on soil biogeochemistry. (Bolan et al. 2011; Chantigny 2003; Kalbitz et al. 2000). If microorganisms break down the carbon (C) stored away in permafrost, this opens a latch into a globally significant C reservoir that might be released into the atmosphere and rivers (as DOM), influencing aquatic chemistry and greenhouse warming (Prater et al. 2007; Schuur et al. 2008; Striegl et al. 2005; Wickland et al. 2006). DOM is made up of humified fractions that are rarely broken down by bacteria and a variety of bioactive compounds that are readily digested by microorganisms (Romero et al. 2017). Bioactive compounds include aliphatic components like amino acids and carbohydrates, along with low molecular weight aromatic fractions such as fulvic acids. Additionally, the humified fractions are composed of intricate aromatic structures that originate from the decomposition of lignocellulosic polymers (Pan et al. 2017; Vázquez et al. 2016). Due to these intricate components, soil formation, mineral weathering, biological activity, and the movement of metals and organic contaminants are all more susceptible to DOM than to total organic matter. (Chantigny 2003; Gao et al. 2017; Kalbitz et al. 2000; Wei et al. 2014). To date, most studies of DOM in boreal and arctic areas focus on rivers and streams (Amon et al. 2012; O'Donnell et al. 2014; Raymond et al. 2007). The expansion of thermokarst lakes caused by permafrost degradation has significantly changed the chemical composition of sediments and pore water (Manasypov et al. 2021). The overall transfer of DOM from terrestrial ecosystems to surface waters is significant; however, few studies have investigated the composition and sources of DOM in soils or traced the DOM signal in permafrost before it is diminished through dilution, photodegradation, and/or microbial consumption of carbon (Abbott et al. 2014; Mann et al. 2015). Furthermore, examining the makeup, source of carbon, and possible lability of DOM is crucial for comprehending the extent of potential feedbacks to climate change resulting from permafrost melting.

The decrease in the extent of permafrost impacts surface hydrology and significantly changes the quantity and types of DOM and nutrients that transition from land‐based ecosystems to aquatic environments. For instance, research on ice‐rich Yedoma permafrost reveals that the ancient organic material trapped within permafrost holds elevated concentrations of low aromaticity small molecular‐weight organic acids, making it readily biodegradable. Consequently, it is quickly utilized by microorganisms and emitted into the atmosphere following thawing (Vonk et al. 2013). Previous studies have assessed the potential positive feedbacks of permafrost thaw on climate change, but research on permafrost DOM composition and provenance remains scarce. In the context of peatland permafrost, how DOM composition changes in different permafrost zones remains underexplored.

The current research combined the analysis of ultraviolet–visible (UV–Vis) spectra with parallel factor (PARAFAC) evaluation of fluorescence excitation‐emission matrix (EEM) spectrophotometry to assess the vertical properties of DOM under continuous permafrost, discontinuous permafrost, isolated patches permafrost at Great Xing'an Mountains. The specific aims of this research were: (i) to measure the variation in soil dissolved organic carbon (DOC) across different thaw gradients, (ii) to examine the vertical characteristics of DOM within the 0–50 cm soil layer, and (iii) to enhance the understanding of DOM properties in relation to varying permafrost thaw gradients.

## 2 Materials and Methods

### 2.1 Study Site

**FIGURE 1:**
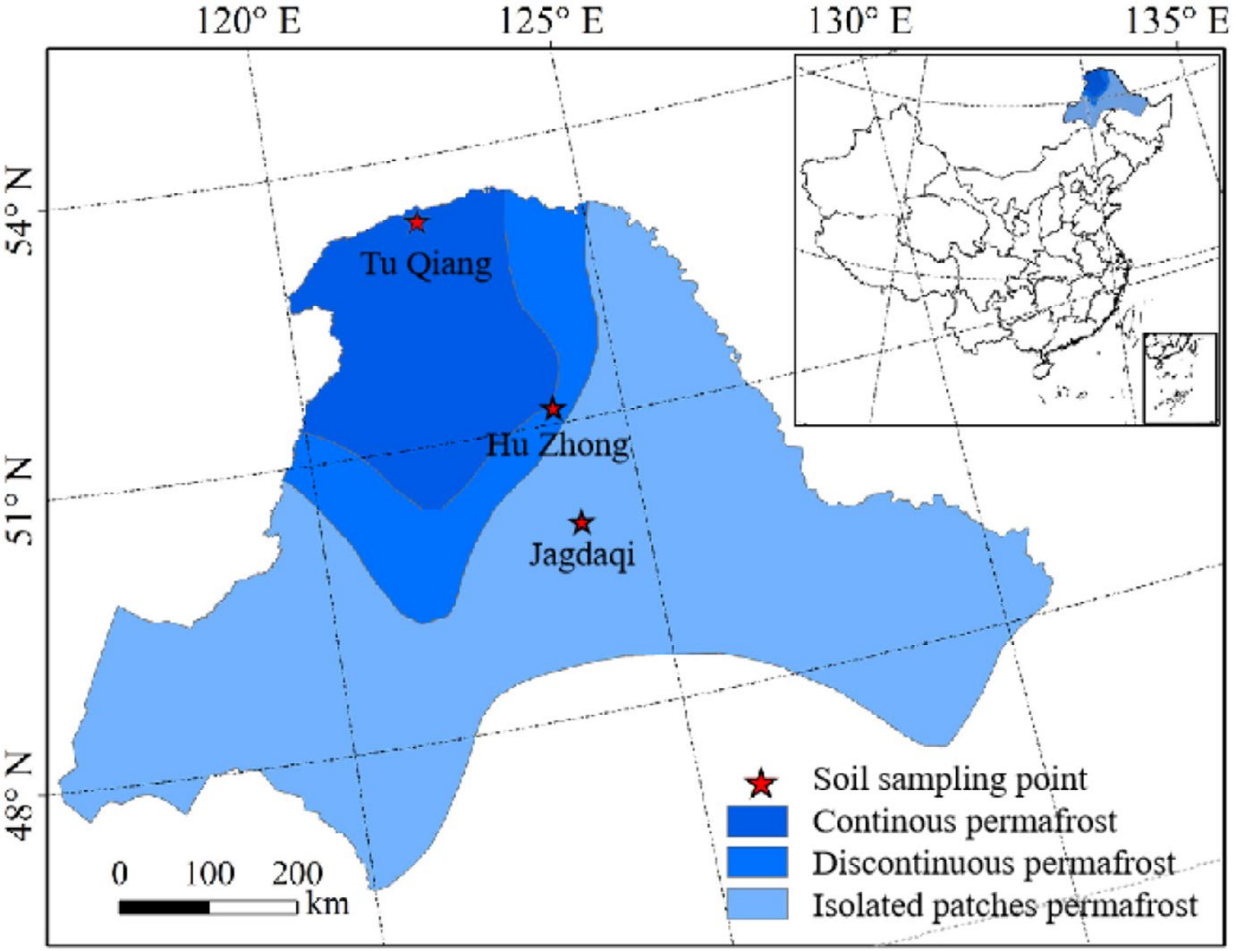
Sampling sites in permafrost zones of Northeast China.

Located in the Great Xing'an Mountains (52°200 N, 124°420 E; Figure 1), the study site extends from the southern to the north, with permafrost transitioning from isolated patches permafrost to a thawing zone resembling islands and eventually large continuous permafrost (Jin et al. 2021). This area has a characteristic temperate continental climate, characterized by warm and humid summers and extended cold winters, with an average yearly temperature of 2°C and significant local climate differences. Peatland soils and permafrost were present at all study sites and had the same vegetation type. The shrub layer is mainly composed of *Vaccinium uliginosum* , *Rhododendron dauricum* and *Ledum palustre* , while the herbaceous layer is mainly characterized by *Carex Aspen* and *Eriophorum vaginatum* . By conducting on site sampling and inspections, we classified the study zones into three levels of permafrost gradient (Table 1). Criteria for the classification of frozen soil degradation stages based on the IPCC Sixth Evaluation Report (Hock et al. 2019): Continuous permafrost (TQ): annual average ground temperature < −3°C, frozen soil coverage > 90% Discontinuous permafrost (HZ): annual average ground temperature −1°C to −3°C, coverage rate 50%–90%. Isolated patches permafrost (JGDQ): annual average ground temperature > 0°C, coverage rate < 10%.

**TABLE 1:** Sampling point location information and active layer thickness.

Location	Coordinates	Active layer thickness	Permafrost zone
Tu Qiang (TQ)	122**°**46′25″ 52**°**55′01″	50 ± 6 cm	Continuous permafrost
Hu Zhong (HZ)	123**°**08′44″ 52**°**06′06″	60 ± 3 cm	Discontinuous permafrost
Jiagedaqi (JGDQ)	124**°**13′21″ 50**°**28′07″	80 ± 5 cm	Isolated patches permafrost

### 2.2 Soil Sampling and Preparation

By conducting on‐site sampling and inspection in the research site, we categorized the study zones into three gradients of permafrost: Tu Qiang (continuous permafrost zone); Hu Zhong (discontinuous permafrost zone); and Jiagedaqi (isolated patches permafrost zone), there was a significant difference in the thickness of the active soil layer between the three sample sites. Sampling locations were selected randomly during September of 2023, as this is when the active layer reaches its maximum depth. The maximum thawing depths of the three types of permafrost zones were measured through on‐site inspections conducted at the end of September. The measured depths were as follows: TQ: 50 ± 6 cm, HZ: 60 ± 3 cm, and JGDQ: 80 ± 5 cm. For this study, soil samples were gathered from depths of 0–50 cm. Three parallel sample plots were selected in each area, with a distance of approximately 20 m between each profile. In each plot, five soil samples were gathered from quadrats measuring 1 m × 1 m, with four samples extracted from the corners and one from the center. These were then combined to form a composite sample. From each area, three samples were collected and placed in an incubator for subsequent analysis. To maintain the samples' integrity during transport, segmentation of the soil was performed at the sampling location, after which the samples were bagged and kept in a refrigerator at −20°C. Beforehand, following the removal of debris, surface vegetation, and plant roots by hand. To conduct the analysis, soil samples were first air‐dried at room temperature for a period of 7 days before being sieved through a 2 mm mesh. Following this, the samples were stored at a temperature of 4°C until further evaluation.

### 2.3 Water‐Extractable DOM Extraction

The extraction of soil DOM utilized water‐soil agitation. To elaborate, soil specimens were immersed in deionized water with a solid/liquid ratio of 1/6 (w/v) and agitated at a speed of 180 rpm for a duration of 24 h. The procedure is outlined as follows: the mixture was subjected to centrifugation at 8000 g for 10 min, and this was followed by filtration through 0.45 μm cellulose acetate membrane filters. The resultant filtrate was preserved at 4°C for later analysis. This entire procedure was carried out three times to ensure accuracy. The Multi N/C 3100 instrument (Analytik‐Jena, Jena, Germany) was utilized to measure DOC, the instrument measurement accuracy is 0.01 mg/kg, but for the sake of simplifying the displayed value, it has been rounded.

### 2.4 UV–Vis and Fluorescence Spectra

The absorbance in the UV–Vis spectrum was recorded over a scanning range of 250 to 800 nm with a UV spectrophotometer (Thermo, CARY4000 UV–Vis, USA). This research employed various significant parameters from the UV–Vis spectra (Green and Blough 1994; Helms et al. 2008; Weishaar et al. 2003). Particularly, the specific ultraviolet absorbance measured at 254 nm (SUVA) is determined by calculating the ratio of A254 to the concentration of DOC (SUVA_254_). Generally, a greater SUVA_254_ suggests a higher level of aromaticity in DOM (Weishaar et al. 2003). The slope ratio (*S*_R_), which is generally inversely correlated with the molecular weight of DOM (Amon et al. 2012), was calculated by comparing the slope between the wavelengths of 275–295 nm to that of 350–400 nm, in accordance with the methodology outlined by Helms et al. (2008).

Weight average molecular weight index (WAMW) as follows (Chin et al. 1994):

(1) $$\text{WAMW}=\left(3.99\times \mathrm{MA}\right)+490$$

(2) $$\mathrm{MA}\;\text{is Molar Absorptivity}\frac{\left(A280\times 1000\right)}{\left(\frac{\mathrm{DOC}}{12}\right)}$$

WAMW for inference of carbon aromaticity, molecular weight and DOM source (Chin et al. 1994).

The fluorescence intensity was recorded using a fluorescence spectrometer (Hitachi, F‐7000, Japan) at excitation wavelengths ranging from 230 to 500 nm and emission wavelengths between 250 and 550 nm, facilitating the creation of the 3D‐EEM. The intervals for scanning the excitation wavelengths were set at 5 nm, while for the emission wavelengths, the intervals were 3 and 2 nm, respectively. The scanning speed was set at 6000 nm/min, and absorbance measurements were utilized to correct the internal filter effects of all EEMs. Additionally, we addressed the majority of Raman scatter effects by subtracting the Milli‐Q blank. Fluorescence index (Shafiquzzaman et al. 2014), a metric used to identify the source of DOM. A value of around 1.8 indicates DOM derived from microbial sources, while approximately 1.2 suggests terrestrial‐derived DOM, primarily reflecting plant‐dominated sources. This index is determined by calculating the emission intensity at 470 nm and dividing it by the intensity at 520 nm, with an excitation wavelength set at 370 nm (Cory and McKnight 2005; Fellman et al. 2010). The humification index (HIX), which indicates the relative quantity of humic substances, was determined by calculating the area under the emission spectra at wavelengths of 435–480 nm and dividing it by the area within the emission range of 300–345 nm (Ohno 2002; Parlanti et al. 2000). The Biological Index (BIX), which indicates the fraction of fresh autochthonous DOM, was calculated by taking the ratio of the fluorescent intensity measured at 380 nm to the highest intensity recorded in the emission range of 420–435 nm (Wilson and Xenopoulos 2009). Typically, when soil‐derived DOM was analyzed using BIX, it was often correlated with FI and could also suggest microbial contributions to DOM (Gao et al. 2017; Wang et al. 2016).

### 2.5 Statistical Analysis

Statistical analyses were conducted utilizing SPSS version 20.0 (SPSS Inc., Chicago, IL, USA). Results are shown as means and standard deviations. To examine the variations in DOC content across various soil depths and permafrost types, one‐way ANOVA and least‐significant difference (LSD) tests were employed (*p* < 0.05), following the assessment that the data typically satisfied the criteria for normal distribution and variance homogeneity. The Tukey honestly significant difference (Tukey HSD) test was applied to evaluate the differences in soil fluorescence characteristics. PARAFAC breaks down the EEM data set into multiple trilinear components with the aid of MATLAB software R2019b and the DOM Fluor toolbox (Ding et al. 2020). Furthermore, principal component analysis (PCA) was employed to examine the characteristics of the fluorescence index and soil composition. The Pearson correlation coefficients (*r*) among various DOM parameters were computed at a significance level of 5% (*p* < 0.05), 1% (*p* < 0.01) or 0.1% (*p* < 0.001).

## 3 Results

### 3.1 Changes in Soil DOC

**FIGURE 2:**
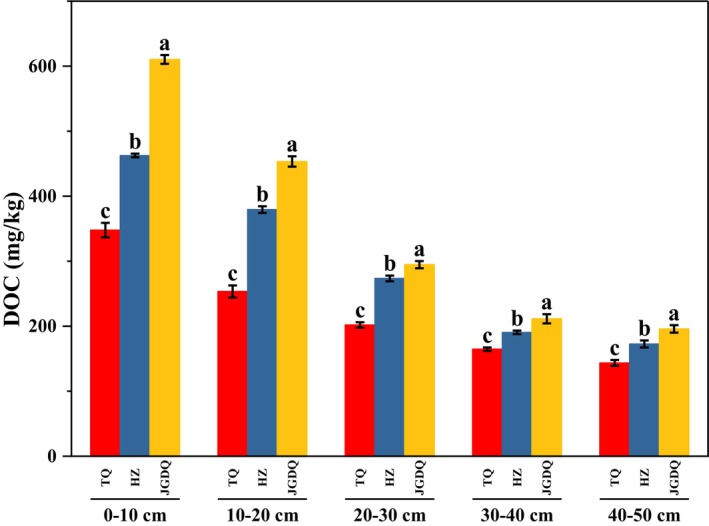
Variations of dissolved organic carbon content in 0–50 cm soil under different permafrost zones (continuous permafrost, discontinuous permafrost, isolated patches permafrost) (the same letter indicates no significant difference among groups, while different letters indicate significant differences, with a significance level of 0.05.).

Permafrost has been identified in all three types of permafrost zones, where numerous freeze–thaw cycles have influenced the interface between the active layer and the permafrost. The average DOC levels across different permafrost zones were assessed from samples taken from the 0–50 cm depth of soil, and the findings were ordered as follows: TQ (143–347 mg/kg); HZ (172–462 mg/kg); JGDQ (195–610 mg/kg) (Figure 2, Table S1). The DOC value decreases as the soil depth increases, while the DOC value gradually increases from the continuous frozen soil zone to the island patches permafrost zone. The numerical differences of DOC in the three types of permafrost zones are significant (*p* < 0.05).

### 3.2 Identification of DOM Components

The spectral loadings, EEMs, and peak locations of fluorescence for the five identified components are shown in Table 2, which can be utilized for the qualitative analysis of DOM components. Component 1 (C1) was characterized by high molecular weight and low redox activity, resembling UVC humic‐like structures (Ishii and Boyer 2012). Component 2 (C2) was represented fluorescent groups similar to humus‐like fluorescent groups derived from animal manure fertilizers from human activities and/or agricultural fields (Stedmon and Markager 2005). Component 3 (C3) was characterized by the presence of fulvic acid‐like compounds, which are typically associated with carboxyl and carbonyl functional groups (D'Andrilli et al. 2013; Korak et al. 2015). Research indicates that as the emission wavelength increases, there is a corresponding rise in the quantity of aromatic, conjugated, and high molecular weight humic‐like structures (Lichtman and Conchello 2005). In comparison to C1, Component 4 (C4) consisted of larger and more hydrophobic compounds, as evidenced by the extended peak of excitation and emission wavelengths. Therefore, C4 can be recognized as a terrestrial UVA humic‐like substance characterized by high molecular weight, aromatic properties, and widespread distribution in wetlands and forest ecosystems (Fellman et al. 2010; Ishii and Boyer 2012). Tryptophan‐like molecules were identified as Component 5 (C5) (Stedmon and Markager 2005). This particular type of component is associated with microbial decomposition and typically features a low molecular weight and a simple structure (Coble et al. 1998; Parlanti et al. 2000). The findings revealed that the principal elements of DOM consisted of humic acid‐like (C1–C4) and protein‐like (C5) substances, aligning with previous research outcomes. These components are commonly recognized in the DOM from both aquatic and soil ecosystems and signify typical organic matter (Gu et al. 2019).

**TABLE 2:** The locations of excitation‐emission matrices (EEMs), representative EEMs, and the fluorescence spectral loadings for the five fluorescence components determined through PARAFAC modeling across various soil samples from distinct permafrost zones (continuous permafrost, discontinuous permafrost, isolated patches permafrost).

Component	Approximate EEMs location	Fluorescent compounds	EEMs	Spectral loadings
C1	Ex: 270 (370) nm Em: 455 nm	UVC humic‐like high molecular weight and aromatic humic acid	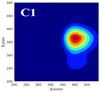	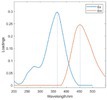
C2	Ex: 330 nm Em: 406 nm	Humic‐like high molecular weight, syringaldehydlike (byproducts of lignin breakdown), associated with high DOM contents	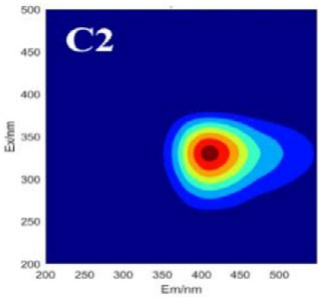	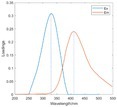
C3	Ex: 260 nm Em: 440 nm	Fulvic acid‐like lower molecular weight, fluorescence resembles fulvic acid	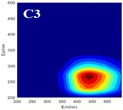	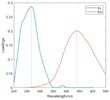
C4	Ex: 290 (403) nm Em: 500 nm	UVA humic‐like high molecular weight humic, related to degree of humification	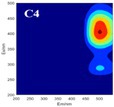	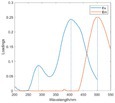
C5	Ex: 350 nm Em: 380 nm	Tyrosine‐like—Amino acid, soluble bound microbial DOM, may indicate more degraded peptide material	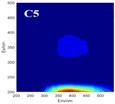	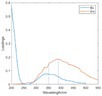

### 3.3 Relative Distribution of DOM Components

**FIGURE 3:**
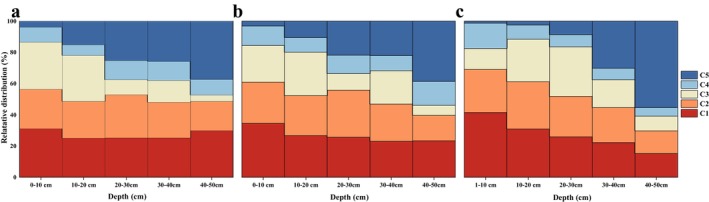
Relative distributions of different fluorescence components (C1: UVC humic‐like; C2: humic‐like; C3: fulvic acid‐like; C4: UVA humic‐like; C5: tyrosine‐like) calculated by PARAFAC modeling in 0–50 cm under different permafrost zones (a: continuous permafrost; b: discontinuous permafrost; c: isolated patches permafrost).

The relative distribution of the five components identified through PARAFAC modeling is illustrated in Figure 3, Table S2. Overall, the analysis revealed that the proportion of humic acid‐like substances (C1–C4) diminished with increasing soil depth. In all samples, C1 content was similar to C2 content and had a tendency to change in a similar way. UVC humic acid (C4) analogs were the lowest, and the content of C3 did not show a clear pattern of change, but it was low in the 40–50 cm soil layer. In addition, the contribution of C5 to DOM fluorescence in the isolated patches of permafrost evidently differed from that of the other permafrost zones. At the same time, during the transition from continuous permafrost to isolated patch permafrost, the C5 content in the 40–50 cm soil layer increased significantly. Moreover, in isolated patches of permafrost, the proportion of C5 in the 40–50 cm soil layer exceeds 50%.

### 3.4 DOM Quality Indexes

**FIGURE 4:**
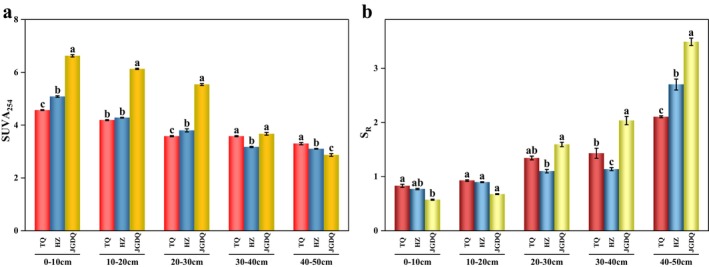
Variations of SUVA_254_ (a), *S*_R_ (b) under different permafrost zones (continuous permafrost, discontinuous permafrost, isolated patches permafrost) (the same letter indicates no significant difference among groups, while different letters indicate significant differences, with a significance level of 0.05.).

The spectral parameters in the UV–Vis range for SUVA_254_ and *S*_R_ were employed to investigate the characteristics of DOM components released from various soils (Figure 4). The SUVA_254_ values at all permafrost gradients were found to be highest at a depth of 0–10 cm, subsequently decreasing with increasing soil depth, while the surface soil SUVA_254_ progressively rose from the continuous permafrost zones to the island patches of permafrost zones (Figure 4a). In different permafrost zones, the *S*_R_ value shows an increasing trend from top to bottom (Figure 4b). At the same time, we observe that the size of the *S*_R_ value at 40–50 cm is (JGDQ > HZ > TQ).

To better elucidate the source and quality of DOM, several optical indices such as FI, HIX, and BIX are presented in Figure S1. In the TQ, HZ and JGDQ samples, there were no significant differences in the BIX, FI and HIX values among the permafrost types. The average BIX values were 0.82 (TQ), 0.54 (HZ) and 0.54 (JGDQ) respectively; the average FI values were 1.73 (TQ), 1.76 (HZ) and 1.82 (JGDQ); the average HIX values were 0.83–0.94 (TQ), 0.89–0.96 (HZ) and 0.79–0.99 (JGDQ). Statistical analysis confirmed that there were no significant differences (*p* > 0.05).

## 4 Discussion

### 4.1 Variation in DOC Content

Previous studies have shown that DOC content in shallow soils of perennial permafrost peatlands is large and tends to decrease and then increase from top to bottom (Lu et al. 2022). In this study, the DOC value decreases with increasing depth in all permafrost zones; the lack of an increase in DOC values in the subsoil can be attributed to the fact that the 40–50 cm soil layer has not yet reached the permafrost interface and has not developed carbon enrichment (Lim et al. 2021). The DOC content drops rapidly at 10–20 cm (Figure 2), which indicates that a substantial amount of organic matter has accumulated in the topsoil, where microorganisms consume considerable quantities of plant litter, roots, and other decomposed substances, resulting in the release of abundant DOC (Scott and Rothstein 2014). In addition, root exudation is also likely responsible for higher levels of surface DOC through direct excretion of fresh DOC into the soil. However, the DOC content at depths of 30–50 cm showed minimal variation. This phenomenon can be attributed to the interaction of several factors, including the leaching and deposition of sediments, microbial activity within the soil, adsorption by mineral clays, and the reduction of plant roots in deeper soil layers, all of which collectively contribute to carbon fixation (Scott and Rothstein 2014; Wang et al. 2015).

The DOC value shows an increasing trend from the continuous permafrost zone to the island patches permafrost zone. The mean annual surface temperature increased significantly at a rate of 0.008°C per year from 2000 to 2020, while the southern boundary of the permafrost zone shifted north by 0.1° to 1° (Che et al. 2023). The spatial variability leads to a step change in regional temperatures in different permafrost zone, which results in significant permafrost degradation (Zhang et al. 2005) and deepening of the active permafrost layer. At the same time, DOC content directly affects microbial activity (Weiss et al. 2016), thus changing the effectiveness of soil nutrients and increasing the potential for carbon decomposition. From this we speculate that as the permafrost boundary moves northward, there will be more carbon emissions.

### 4.2 Characteristics of DOM

The relative distribution of the five fluorescent components of DOM is illustrated in Figure 3. This suggests that the DOM present in the topsoil primarily originates from humic acid‐like substances. In contrast, the DOM found in the subsoil is predominantly a result of microbial degradation, with microbial products progressively increasing as soil depth increases (Kaiser et al. 2004; Vázquez et al. 2016). As shown in Figure S2, the Pearson correlation analyses between humic acid (C1, C2, C3) and C5 in different permafrost zones also showed a noteworthy negative correlation (*p* < 0.05). This finding implies the possible existence of a transformational mechanism linking high molecular weight humic acid‐like substances and tryptophan‐like compounds. It is worth noting that the tryptophan substance in the surface soil gradually decreases from continuous permafrost zone to island patches permafrost zone (Figure 3). This may be due to the fact that the environment (high temperature, high moisture content) in island patches permafrost zones is more conducive to microbial activities, which enhances the ability of microorganisms to gradually assimilate small molecule tryptophan analogues during long‐term accumulation, and the release of humic acid analogues with larger molecular weights (Li et al. 2019). Experimental research shows that in permafrost wetland, the warming and decomposition of surface soil mainly focuses on CO_2_. The cumulative mineralization amount of SOC shows an increasing trend from continuous permafrost area to sporadic patch permafrost area (Dong et al. 2023). This study studied the percentages of humic acid and protein substances in permafrost DOM under different melt gradients, the decrease in humic acid content in shallow soil, and the increase in tryptophan or lysine content in the subsoil, which is related to decomposition of plants and algae and microbial metabolism are closely related. Earlier research has indicated that substances resembling humic acid, which possess large molecular sizes, conjugated aromatic groups, and intricate structures, appear to be less readily utilized by microorganisms in comparison to protein‐like substances that have simpler structures (Guo et al. 2012; Pan et al. 2017; Vázquez et al. 2016). The results of ^1^H‐NMR indicate that the overall content of aromatic substances is continuous > discontinuous > isolated patches permafrost zones (Figure S3). In the continuous permafrost zone, the proportion of stable macromolecular structure aromatics in DOM is higher (about 10%), while in the isolated patches permafrost zone, the proportion of aromatic substances in deep soil is only 3.5%. The continuous permafrost zone enriches stable components (MDLT accounts for 35%–49%), and its long‐chain lipid structure requires higher energy to break. Therefore, it is inferred that DOM under the continuous permafrost zone has higher stability. In addition, the content of carbohydrates in the isolated patches permafrost zone is relatively high (26.3%–33.2%), while in the continuous permafrost zone, it is only 16.6%–23.6% (Table S3). The higher content of carbohydrates will also reduce the stability of DOM. Based on the above results, we assume that the northward movement of the permafrost boundary will enhance the microbial availability of surface DOM and enhance the humification of DOM, reducing the stability of DOM.

### 4.3 The Quality of DOM in Soil

In this research, the levels of SUVA 254 in the topsoil were found to be significantly greater compared to those in the subsoil across various permafrost gradients (Figure 4a). These findings suggest that as soil depth increases, the humification of soil humus decreases, a trend corroborated by the variations observed in the HIX index (Figure S1). Throughout all permafrost zones, high molecular weight and highly aromatic hydrophobic components were identified in the topsoil. Conversely, in the subsoil, hydrophilic components steadily accumulated, a process attributed to the selective adsorption of hydrophobic DOM with high aromatic characteristics and elevated molecular weight by substantial soil particles present in the topsoil (Chantigny 2003; Gao et al. 2017). In addition, according to the value of FI, it can be seen that the endogenous input of DOM to soil is basically the same under different permafrost gradients. The main sources of DOM in these three types of permafrost are phytoplankton and bacteria that consume organic matter from plants (Tang et al. 2019), and there is a trend of transformation from plant sources to microbial sources from top to bottom. From the continuous permafrost area to the island patches permafrost area, there is a slight increase in the plant sources in the surface soil, and the microbial sources in the subsoil are gradually increasing. The value of topsoil HIX is (JGDQ > HZ > TQ), indicating that the surface soil in the island frozen soil area has a higher degree of humification. It is worth noting that the degree of soil humification in the three permafrost zones shows a gradual decrease from top to bottom (Figure S1c). This suggests that the DOM across various gradients exhibits increased humification within the topsoil, and that microorganisms gradually decompose and transform the accumulated humus as soil depth increases. This is further supported by the strongly significant positive correlation (*p* < 0.01) observed between the HIX index and the SUVA_254_ index across the three permafrost zones, as shown in Figure S2. BIX is an indicator of the biological activity of DOM. The primary source of organic matter in peatlands is the gradual accumulation and destruction of leaf litter over time. Consequently, when considering various frozen soil gradients, the amount of local carbon entering the surface soil is minimal in the short term, and the production of newly formed DOM is relatively limited (Ros et al. 2010; Wilson and Xenopoulos 2009). Higher BIX values indicate that biogenic sources such as algae and bacteria contribute more to DOM (Wilson and Xenopoulos 2009). Changes in BIX values indicate that the deeper the soil, the more the composition of DOM is affected by internal processes such as algae and bacterial activities, and the newly generated DOM due to microbial degradation gradually increases. According to the results, it is inferred that the contribution of DOM derived from microorganisms in the subsoil is higher.

**FIGURE 5:**
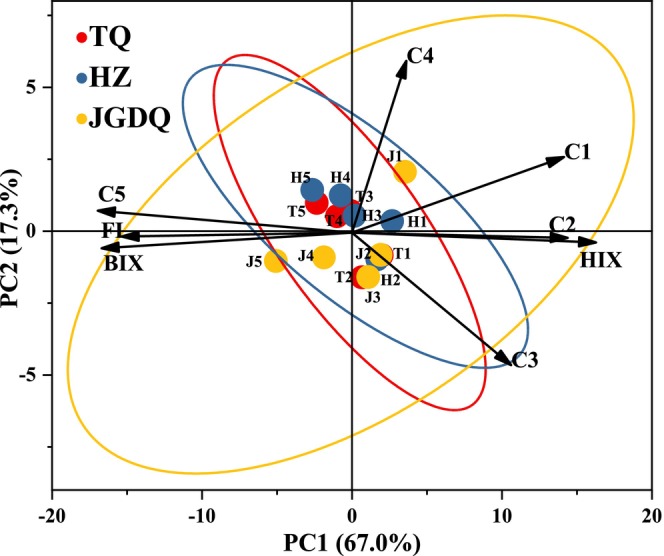
Principal component analysis (PCA) of soil DOM composition.

Principal Component Analysis indicated that the first and second principal components (labeled as PC1 and PC2) explained 67.0% and 17.3% of the variation, respectively (Figure 5). PC1 showed a significant positive relationship with C2, C1, and HIX, while it displayed a notable negative relationship with BIX, FI, and C5. In contrast, PC2 demonstrated a positive correlation with the C4 index and a negative correlation with C3. We observed significant differences in the spatial distribution of soil DOM in PCA space across various types of permafrost zones. This finding indicates that the type of permafrost plays a critical role in the formation and evolution of DOM. The properties of DOM in the continuous and the discontinuous permafrost zone were found to be similar; however, marked differences were noted between these areas and the DOM in the island patches permafrost zone. This change may significantly affect the soil ecosystem's capacity to retain OM and its evolutionary characteristics, particularly concerning soil depth and types of permafrost. With the reduction in permafrost coverage, the sources of soil DOM have undergone notable changes. In shallow soil layers, DOM is closely associated with terrestrial aromatic humic compounds and lignin, while in deeper soil layers, it is significantly influenced by microbial metabolism (Shafiquzzaman et al. 2014). In addition, the possible conversion mechanism between humic acid substances and protein substances needs further study. With the changing types of permafrost, the stability of deep soil DOM significantly decreases. Revealing the spatial variations in DOM will enhance our comprehensive understanding of the dynamic characteristics and ecological functions of soil organic matter.

### 4.4 The Effect of Permafrost Gradient on the DOM Characteristics

**FIGURE 6:**
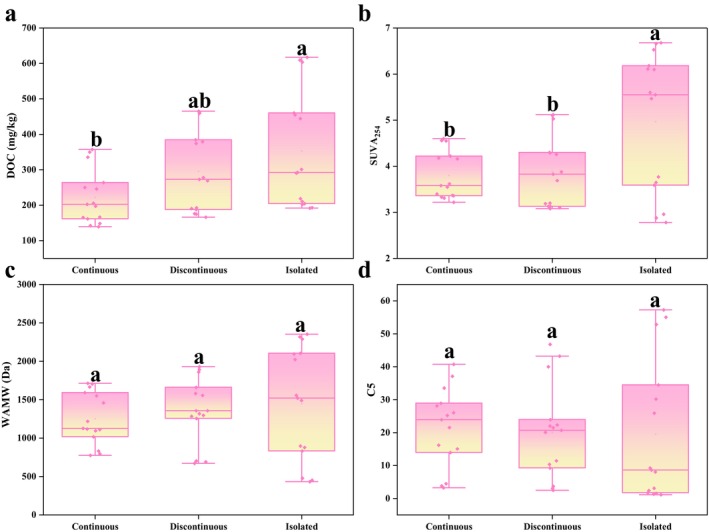
Dependence of DOC (a) concentration, optical parameters SUVA (b), WAMW (c) and C5 (d) in different permafrost zones (continuous permafrost, discontinuous permafrost, isolated patches permafrost) (the same letter indicates no significant difference among groups, while different letters indicate significant differences, with a significance level of 0.05.).

To assess the dynamics of DOM across the permafrost gradient, we calculated the average of the pertinent parameters for each area (Figure 6). The largest DOC appears in the island patches permafrost zone. The thickness of the active layer in isolated patches permafrost zone exceeds that of the other two types of permafrost, resulting in a more substantial upward transport of ancient peat leachate to the upper layers (Raudina et al. 2017). The study found that the aromaticity of DOM in the surface soil of island patch permafrost zones is higher than that in the other two types of permafrost zones, a characteristic likely associated with the unique environmental conditions and plant communities present in this region. In contrast, the DOM in the deeper soil layers demonstrates a higher protein content and the lowest molecular weight, suggesting a more active organic matter decomposition process occurring within this soil layer. The molecular weight, aromaticity, and composition of DOM vary with the type of permafrost.

Taking advantage of the fact that sampling points in this research are located in different types of permafrost zones respectively (Guo et al. 2023), we can use space instead of time methods to predict possible changes in DOM composition and quality as the climate warms and the permafrost boundary moves northward. The possible evolution of the chemical composition of lake water for different environmental components has been successfully predicted using spatiotemporal alternatives in western Siberia (Manasypov et al. 2014). Our findings suggest that in peatlands, microorganisms utilize humic DOM derived from plants as an energy source, transforming these materials into compounds characterized by lower molecular weight and aromatic features. When the continuous permafrost zone transitions to the island patches permafrost zone, the DOC content in the soil increases, and the resources available to microorganisms increase, which may in turn lead to an increase in carbon release in the island patches permafrost zone. We speculate that the molecular weight of DOM in the subsoil of continuous/discontinuous permafrost zones will decrease and the potential bioavailability will increase under climate warming scenarios.

## 5 Conclusions

This research systematically reveals the distribution characteristics of DOM in the 0–50 cm soil layer of peatlands across continuous, discontinuous, and island patches permafrost zones in Daxing'anling. The results indicate that permafrost types significantly influence the content of soil DOC, the sources of DOM, and its bioavailability. Firstly, the increase in DOC concentration from continuous permafrost zones to island patches permafrost zones indicates that the northward migration of the permafrost boundary will accelerate the release of surface organic carbon. Notably, components C1–C4 dominate the composition of surface soil DOM, while C5 is more prevalent in deeper DOM. The significant negative correlation (*p* < 0.05) between the accumulation of microbial metabolites and humic acid substances suggests that the dynamic transformation of the humic‐protein components of DOM is a core mechanism driving carbon migration and decomposition release. Secondly, the surface DOM in island patches permafrost zone exhibits relatively high aromaticity and humification, likely due to the high‐activity environment promoting the re‐synthesis of humification products from plant residues. However, with increasing depth, the molecular weight of DOM significantly decreases, the proportion of C5 increases, and the contribution of DOM metabolized by subsoil microorganisms enhances, indicating that the potential bioavailability of deep DOM improves following the northward movement of the permafrost boundary. Principal component analysis further confirms the significant influence of permafrost types on the composition of DOM. This study provides a crucial scientific basis for evaluating the stability of soil DOM across various permafrost zones and the carbon‐climate feedback mechanisms in the context of climate warming. Future research should integrate stable isotope tracing (such as δ^13^C) with metagenomics technology to quantify the microbial mechanisms driving the dynamic transformation of DOM, thereby enabling a more accurate assessment of the response threshold of the permafrost carbon pool to global changes.

## Author Contributions

**Siyuan Zou:** conceptualization (equal), data curation (equal), formal analysis (equal), validation (equal), writing – original draft (equal). **Xiaodong Wu:** conceptualization (equal), formal analysis (equal), investigation (equal), writing – review and editing (equal). **Jiawei Zhang:** conceptualization (equal), methodology (equal), visualization (equal). **Nannan Zhang:** investigation (equal), methodology (equal). **Xiangwen Wu:** conceptualization (equal), data curation (equal), formal analysis (equal), funding acquisition (equal), visualization (equal), writing – review and editing (equal). **Shuying Zang:** conceptualization (equal), data curation (equal), funding acquisition (equal), writing – review and editing (equal).

## Conflicts of Interest

The authors declare no conflicts of interest.

## Supporting information


Appendix S1.

## Data Availability

The data that supports the findings of this study are available in the Supporting Information of this article.

## References

[ece371667-bib-0001] Abbott, B. W. , J. R. Larouche , J. B. Jones Jr. , W. B. Bowden , and A. W. Balser . 2014. “Elevated Dissolved Organic Carbon Biodegradability From Thawing and Collapsing Permafrost.” Journal of Geophysical Research: Biogeosciences 119: 2049–2063.

[ece371667-bib-0002] Amon, R. , R. M. W. Amon , A. J. Rinehart , et al. 2012. “Dissolved Organic Matter Sources in Large Arctic Rivers.” Geochimica et Cosmochimica Acta 94: 217–237.

[ece371667-bib-0003] Biskaborn, B. K. , S. L. Smith , J. Noetzli , et al. 2019. “Permafrost Is Warming at a Global Scale.” Nature Communications 10: 264. 10.1038/s41467-018-08240-4 PMC6335433 30651568

[ece371667-bib-0004] Bolan, N. S. , D. C. Adriano , A. Kunhikrishnan , T. James , R. McDowell , and N. Senesi . 2011. “Dissolved Organic Matter: Biogeochemistry, Dynamics, and Environmental Significance in Soils.” Advances in Agronomy 110: 1–75.

[ece371667-bib-0005] Chantigny, M. H. 2003. “Dissolved and Water‐Extractable Organic Matter in Soils: A Review on the Influence of Land Use and Management Practices.” Geoderma 113: 357–380.

[ece371667-bib-0006] Che, L. , H. Zhang , and L. Wan . 2023. “Spatial Distribution of Permafrost Degradation and Its Impact on Vegetation Phenology From 2000 to 2020.” Science of the Total Environment 877: 162889. 36933732 10.1016/j.scitotenv.2023.162889

[ece371667-bib-0007] Chen, X. , S. Jeong , C.‐E. Park , et al. 2022. “Different Responses of Surface Freeze and Thaw Phenology Changes to Warming Among Arctic Permafrost Types.” Remote Sensing of Environment 272: 112956.

[ece371667-bib-0008] Chin, Y.‐P. , G. Aiken , and E. O'Loughlin . 1994. “Molecular Weight, Polydispersity, and Spectroscopic Properties of Aquatic Humic Substances.” Environmental Science & Technology 28: 1853–1858. 22175925 10.1021/es00060a015

[ece371667-bib-0009] Coble, P. G. , C. E. Del Castillo , and B. Avril . 1998. “Distribution and Optical Properties of CDOM in the Arabian Sea During the 1995 Southwest Monsoon.” Deep Sea Research Part II: Topical Studies in Oceanography 45: 2195–2223.

[ece371667-bib-0010] Cory, R. M. , and D. M. McKnight . 2005. “Fluorescence Spectroscopy Reveals Ubiquitous Presence of Oxidized and Reduced Quinones in Dissolved Organic Matter.” Environmental Science & Technology 39: 8142–8149. 16294847 10.1021/es0506962

[ece371667-bib-0011] D'Andrilli, J. , C. M. Foreman , A. G. Marshall , and D. M. McKnight . 2013. “Characterization of IHSS Pony Lake Fulvic Acid Dissolved Organic Matter by Electrospray Ionization Fourier Transform Ion Cyclotron Resonance Mass Spectrometry and Fluorescence Spectroscopy.” Organic Geochemistry 65: 19–28.

[ece371667-bib-0012] Ding, Y. , Z. Shi , Q. Ye , et al. 2020. “Chemodiversity of Soil Dissolved Organic Matter.” Environmental Science & Technology 54: 6174–6184. 32298089 10.1021/acs.est.0c01136

[ece371667-bib-0013] Dong, X. , H. Man , C. Liu , et al. 2023. “Changes in Soil Bacterial Community Along a Gradient of Permafrost Degradation in Northeast China.” Catena 222: 106870.

[ece371667-bib-0014] Fellman, J. B. , E. Hood , and R. G. M. Spencer . 2010. “Fluorescence Spectroscopy Opens New Windows Into Dissolved Organic Matter Dynamics in Freshwater Ecosystems: A Review.” Limnology and Oceanography 55: 2452–2462.

[ece371667-bib-0015] Gao, J. , C. Liang , G. Shen , J. Lv , and H. Wu . 2017. “Spectral Characteristics of Dissolved Organic Matter in Various Agricultural Soils Throughout China.” Chemosphere 176: 108–116. 28259078 10.1016/j.chemosphere.2017.02.104

[ece371667-bib-0016] Green, S. A. , and N. V. Blough . 1994. “Optical Absorption and Fluorescence Properties of Chromophoric Dissolved Organic Matter in Natural Waters.” Limnology and Oceanography 39: 1903–1916.

[ece371667-bib-0017] Gu, W. , S. Huang , S. Lei , J. Yue , Z. Su , and F. Si . 2019. “Quantity and Quality Variations of Dissolved Organic Matter (DOM) in Column Leaching Process From Agricultural Soil: Hydrochemical Effects and DOM Fractionation.” Science of the Total Environment 691: 407–416. 31323586 10.1016/j.scitotenv.2019.07.120

[ece371667-bib-0018] Guo, X. , X. He , H. Zhang , Y. Deng , L. Chen , and J. Jiang . 2012. “Characterization of Dissolved Organic Matter Extracted From Fermentation Effluent of Swine Manure Slurry Using Spectroscopic Techniques and Parallel Factor Analysis (PARAFAC).” Microchemical Journal 102: 115–122.

[ece371667-bib-0019] Guo, Y. , S. Liu , L. Qiu , Y. Wang , C. Zhang , and W. Shan . 2023. “Permafrost Probability Mapping at a 30 m Resolution in Arxan Based on Multiple Characteristic Variables and Maximum Entropy Classifier.” Applied Sciences 13: 10692.

[ece371667-bib-0020] Helms, J. R. , A. Stubbins , J. D. Ritchie , E. C. Minor , D. J. Kieber , and K. Mopper . 2008. “Absorption Spectral Slopes and Slope Ratios as Indicators of Molecular Weight, Source, and Photobleaching of Chromophoric Dissolved Organic Matter.” Limnology and Oceanography 53: 955–969.

[ece371667-bib-0021] Hock, R. , G. Rasul , C. Adler , et al. 2019. “High Mountain Areas.” IPCC Special Report on the Ocean and Cryosphere in a Changing Climate, 131 202.

[ece371667-bib-0022] Hugelius, G. , J. Strauss , S. Zubrzycki , et al. 2014. “Estimated Stocks of Circumpolar Permafrost Carbon With Quantified Uncertainty Ranges and Identified Data Gaps.” Biogeosciences 11: 6573–6593.

[ece371667-bib-0023] Ishii, S. K. , and T. H. Boyer . 2012. “Behavior of Reoccurring PARAFAC Components in Fluorescent Dissolved Organic Matter in Natural and Engineered Systems: A Critical Review.” Environmental Science & Technology 46: 2006–2017. 22280543 10.1021/es2043504

[ece371667-bib-0024] Jin, X.‐Y. , H.‐J. Jin , G. Iwahana , et al. 2021. “Impacts of Climate‐Induced Permafrost Degradation on Vegetation: A Review.” Advances in Climate Change Research 12: 29–47.

[ece371667-bib-0025] Kaiser, K. , G. Guggenberger , and L. Haumaier . 2004. “Changes in Dissolved Lignin‐Derived Phenols, Neutral Sugars, Uronic Acids, and Amino Sugars With Depth in Forested Haplic Arenosols and Rendzic Leptosols.” Biogeochemistry 70: 135–151.

[ece371667-bib-0026] Kalbitz, K. , S. Solinger , J.‐H. Park , B. Michalzik , and E. Matzner . 2000. “Controls on the Dynamics of Dissolved Organic Matter in Soils: A Review.” Soil Science 165: 277–304.

[ece371667-bib-0027] Korak, J. A. , E. C. Wert , and F. L. Rosario‐Ortiz . 2015. “Evaluating Fluorescence Spectroscopy as a Tool to Characterize Cyanobacteria Intracellular Organic Matter Upon Simulated Release and Oxidation in Natural Water.” Water Research 68: 432–443. 25462750 10.1016/j.watres.2014.09.046

[ece371667-bib-0028] Koven, C. D. , E. A. Schuur , C. Schädel , et al. 2015. “A Simplified, Data‐Constrained Approach to Estimate the Permafrost Carbon–Climate Feedback.” Philosophical Transactions of the Royal Society A: Mathematical, Physical and Engineering Sciences 373: 20140423. 10.1098/rsta.2014.0423 PMC4608038 26438276

[ece371667-bib-0030] Li, R. , Q. Wu , X. Li , et al. 2019. “Characteristic, Changes and Impacts of Permafrost on Qinghai‐Tibet Plateau.” Chinese Science Bulletin 64: 2783–2795.

[ece371667-bib-0031] Lichtman, J. W. , and J.‐A. Conchello . 2005. “Fluorescence Microscopy.” Nature Methods 2: 910–919. 16299476 10.1038/nmeth817

[ece371667-bib-0032] Lim, A. G. , S. V. Loiko , D. M. Kuzmina , et al. 2021. “Dispersed Ground Ice of Permafrost Peatlands: Potential Unaccounted Carbon, Nutrient and Metal Sources.” Chemosphere 266: 128953. 33223213 10.1016/j.chemosphere.2020.128953

[ece371667-bib-0033] Lu, B. , L. Song , S. Zang , and H. Wang . 2022. “Warming Promotes Soil CO_2_ and CH_4_ Emissions but Decreasing Moisture Inhibits CH_4_ Emissions in the Permafrost Peatland of the Great Xing'an Mountains.” Science of the Total Environment 829: 154725. 35331769 10.1016/j.scitotenv.2022.154725

[ece371667-bib-0034] Manasypov, R. M. , O. S. Pokrovsky , S. N. Kirpotin , and L. S. Shirokova . 2014. “Thermokarst Lake Waters Across the Permafrost Zones of Western Siberia.” Cryosphere 8: 1177–1193.

[ece371667-bib-0035] Manasypov, R. M. , O. S. Pokrovsky , L. S. Shirokova , et al. 2021. “Biogeochemistry of Macrophytes, Sediments and Porewaters in Thermokarst Lakes of Permafrost Peatlands, Western Siberia.” Science of the Total Environment 763: 144201. 33385841 10.1016/j.scitotenv.2020.144201

[ece371667-bib-0036] Mann, P. J. , T. I. Eglinton , C. P. McIntyre , et al. 2015. “Utilization of Ancient Permafrost Carbon in Headwaters of Arctic Fluvial Networks.” Nature Communications 6: 7856. 10.1038/ncomms8856 PMC4525200 26206473

[ece371667-bib-0037] McGuire, A. D. , D. J. Hayes , D. W. Kicklighter , et al. 2010. “An Analysis of the Carbon Balance of the Arctic Basin From 1997 to 2006.” Tellus B: Chemical and Physical Meteorology 62: 455–474.

[ece371667-bib-0038] O'Donnell, J. A. , G. R. Aiken , M. A. Walvoord , et al. 2014. “Using Dissolved Organic Matter Age and Composition to Detect Permafrost Thaw in Boreal Watersheds of Interior Alaska.” Journal of Geophysical Research: Biogeosciences 119: 2155–2170.

[ece371667-bib-0039] Ohno, T. 2002. “Fluorescence Inner‐Filtering Correction for Determining the Humification Index of Dissolved Organic Matter.” Environmental Science & Technology 36: 742–746. 11878392 10.1021/es0155276

[ece371667-bib-0040] Pan, H. , H. Yu , Y. Song , R. Liu , and E. Du . 2017. “Application of Solid Surface Fluorescence EEM Spectroscopy for Tracking Organic Matter Quality of Native Halophyte and Furrow‐Irrigated Soils.” Ecological Indicators 73: 88–95.

[ece371667-bib-0041] Parlanti, E. , K. Wörz , L. Geoffroy , and M. Lamotte . 2000. “Dissolved Organic Matter Fluorescence Spectroscopy as a Tool to Estimate Biological Activity in a Coastal Zone Submitted to Anthropogenic Inputs.” Organic Geochemistry 31: 1765–1781.

[ece371667-bib-0042] Peng, X. , T. Zhang , O. W. Frauenfeld , et al. 2023. “Active Layer Thickness and Permafrost Area Projections for the 21st Century.” Earth's Future 11: e2023EF003573.

[ece371667-bib-0043] Prater, J. L. , J. P. Chanton , and G. J. Whiting . 2007. “Variation in Methane Production Pathways Associated With Permafrost Decomposition in Collapse Scar Bogs of Alberta, Canada.” Global Biogeochemical Cycles 21: GB4004.

[ece371667-bib-0044] Raudina, T. V. , S. V. Loiko , A. G. Lim , et al. 2017. “Dissolved Organic Carbon and Major and Trace Elements in Peat Porewater of Sporadic, Discontinuous, and Continuous Permafrost Zones of Western Siberia.” Biogeosciences 14: 3561–3584.

[ece371667-bib-0045] Raymond, P. A. , J. W. McClelland , R. M. Holmes , et al. 2007. “Flux and Age of Dissolved Organic Carbon Exported to the Arctic Ocean: A Carbon Isotopic Study of the Five Largest Arctic Rivers.” Global Biogeochemical Cycles 21: GB4011.

[ece371667-bib-0046] Romero, C. M. , R. E. Engel , J. D'Andrilli , et al. 2017. “Bulk Optical Characterization of Dissolved Organic Matter From Semiarid Wheat‐Based Cropping Systems.” Geoderma 306: 40–49.

[ece371667-bib-0047] Ros, G. H. , C. Tschudy , W. J. Chardon , E. J. M. Temminghoff , C. van der Salm , and G. F. Koopmans . 2010. “Speciation of Water‐Extractable Organic Nutrients in Grassland Soils.” Soil Science 175: 15–26.

[ece371667-bib-0048] Ruggiero, L. , A. Sciarra , A. Mazzini , et al. 2023. “Antarctic Permafrost Degassing in Taylor Valley by Extensive Soil Gas Investigation.” Science of the Total Environment 866: 161345. 36603636 10.1016/j.scitotenv.2022.161345

[ece371667-bib-0049] Schuur, E. A. , E. A. G. Schuur , J. Bockheim , et al. 2008. “Vulnerability of Permafrost Carbon to Climate Change: Implications for the Global Carbon Cycle.” Bioscience 58: 701–714.

[ece371667-bib-0050] Scott, E. E. , and D. E. Rothstein . 2014. “The Dynamic Exchange of Dissolved Organic Matter Percolating Through Six Diverse Soils.” Soil Biology and Biochemistry 69: 83–92.

[ece371667-bib-0051] Shafiquzzaman, M. , A. T. Ahmed , M. S. Azam , et al. 2014. “Identification and Characterization of Dissolved Organic Matter Sources in Kushiro River Impacted by a Wetland.” Ecological Engineering 70: 459–464.

[ece371667-bib-0052] Stedmon, C. A. , and S. Markager . 2005. “Resolving the Variability in Dissolved Organic Matter Fluorescence in a Temperate Estuary and Its Catchment Using PARAFAC Analysis.” Limnology and Oceanography 50: 686–697.

[ece371667-bib-0053] Striegl, R. G. , G. R. Aiken , M. M. Dornblaser , P. A. Raymond , and K. P. Wickland . 2005. “A Decrease in Discharge‐Normalized DOC Export by the Yukon River During Summer Through Autumn.” Geophysical Research Letters 32: L21413.

[ece371667-bib-0054] Tang, J. , W. Wang , L. Yang , C. Cao , and X. Li . 2019. “Variation in Quantity and Chemical Composition of Soil Dissolved Organic Matter in a Peri‐Urban Critical Zone Observatory Watershed in Eastern China.” Science of the Total Environment 688: 622–631. 31254828 10.1016/j.scitotenv.2019.06.270

[ece371667-bib-0055] Tarnocai, C. , J. G. Canadell , E. A. G. Schuur , P. Kuhry , G. Mazhitova , and S. Zimov . 2009. “Soil Organic Carbon Pools in the Northern Circumpolar Permafrost Region.” Global Biogeochemical Cycles 23: GB2023.

[ece371667-bib-0056] Vázquez, C. , A. G. Iriarte , C. Merlo , A. Abril , E. Kowaljow , and J. M. Meriles . 2016. “Land Use Impact on Chemical and Spectroscopical Characteristics of Soil Organic Matter in an Arid Ecosystem.” Environmental Earth Sciences 75: 1–13.

[ece371667-bib-0057] Vonk, J. E. , P. J. Mann , S. Davydov , et al. 2013. “High Biolability of Ancient Permafrost Carbon Upon Thaw.” Geophysical Research Letters 40: 2689–2693.

[ece371667-bib-0058] Wang, J.‐J. , R. A. Dahlgren , M. S. Erşan , T. Karanfil , and A. T. Chow . 2016. “Temporal Variations of Disinfection Byproduct Precursors in Wildfire Detritus.” Water Research 99: 66–73. 27135374 10.1016/j.watres.2016.04.030

[ece371667-bib-0059] Wang, Y. , M'a. Shao , C. Zhang , Z. Liu , J. Zou , and J. Xiao . 2015. “Soil Organic Carbon in Deep Profiles Under Chinese Continental Monsoon Climate and Its Relations With Land Uses.” Ecological Engineering 82: 361–367.

[ece371667-bib-0060] Wei, Z. , X. Zhang , Y. Wei , et al. 2014. “Fractions and Biodegradability of Dissolved Organic Matter Derived From Different Composts.” Bioresource Technology 161: 179–185. 24704883 10.1016/j.biortech.2014.03.032

[ece371667-bib-0061] Weishaar, J. L. , G. R. Aiken , B. A. Bergamaschi , M. S. Fram , R. Fujii , and K. Mopper . 2003. “Evaluation of Specific Ultraviolet Absorbance as an Indicator of the Chemical Composition and Reactivity of Dissolved Organic Carbon.” Environmental Science & Technology 37: 4702–4708. 14594381 10.1021/es030360x

[ece371667-bib-0062] Weiss, N. , D. Blok , B. Elberling , et al. 2016. “Thermokarst Dynamics and Soil Organic Matter Characteristics Controlling Initial Carbon Release From Permafrost Soils in the Siberian Yedoma Region.” Sedimentary Geology 340: 38–48.

[ece371667-bib-0063] Wickland, K. P. , R. G. Striegl , J. C. Neff , and T. Sachs . 2006. “Effects of Permafrost Melting on CO_2_ and CH_4_ Exchange of a Poorly Drained Black Spruce Lowland.” Journal of Geophysical Research: Biogeosciences 111: G02011.

[ece371667-bib-0064] Wilson, H. , and M. Xenopoulos . 2009. “Effects of Agricultural Land Use on the Composition of Fluvial Dissolved Organic Matter.” Nature Geoscience 2: 37–41.

[ece371667-bib-0065] Yu, Z. , J. Loisel , D. P. Brosseau , D. W. Beilman , and S. J. Hunt . 2010. “Global Peatland Dynamics Since the Last Glacial Maximum.” Geophysical Research Letters 37: L13402.

[ece371667-bib-0066] Zhang, T. , O. W. Frauenfeld , M. C. Serreze , et al. 2005. “Spatial and Temporal Variability in Active Layer Thickness Over the Russian Arctic Drainage Basin.” Journal of Geophysical Research: Atmospheres 110: D16101.

