# Supplementary material for: Characterizing Soil Dissolved Organic Matter Across a Permafrost Thaw Gradient (Continuous to Isolated Patches) in Northeastern China
Source: Ecol Evol. 2025 Jul 3;15(7):e71667. doi: 10.1002/ece3.71667 (PMC12231039; doi:10.1002/ece3.71667)
Supplement: Supplementary file 1 — Appendix S1. [file ECE3-15-e71667-s001.docx]

**Supplementary Materials**

**Table S1.** DOC and spectroscopic indices in all sanmples at soil depths of 0-10cm, 10-20cm, 20-30cm, 30-40cm and 40-50cm at the continuous permafrost zone (TQ), discontinuous permafrost zone (HZ), isolated patches permafrost zone (JGDQ) (Mean ± SD).

| Location | Soil depth | DOC (mg/kg) | SUVA_254_ | S_R_ | HIX | BIX | FI | WAMW |
| --- | --- | --- | --- | --- | --- | --- | --- | --- |
|  | 0-10 cm | 347.83±11.15 | 4.50±0.13c | 0.83±0.05a | 0.94±0.02a | 0.75±0.03a | 1.53±0.06a | 1694±25c |
|  | 10-20 cm | 253.43±9.43 | 4.15±0.03b | 0.93±0.02a | 0.93±0.01a | 0.78±0.05a | 1.65±0.05a | 1534±67b |
| TQ | 20-30 cm | 201.95±4.19 | 3.58±0.04b | 1.34±0.06ab | 0.92±0.01a | 0.80±0.05a | 1.76±0.06a | 1077±53c |
|  | 30-40 cm | 164.75±2.63 | 3.58±0.03a | 1.43±0.16b | 0.86±0.02a | 0.83±0.06a | 1.82±0.08a | 1152±58b |
|  | 40-50 cm | 143.55±4.42 | 3.30±0.08a | 2.10±0.03c | 0.83±0.03a | 0.97±0.07a | 1.84±0.06a | 801±29a |
|  | 0-10 cm | 462.58±2.83 | 5.05±0.10b | 0.77±0.02ab | 0.96±0.01a | 0.65±0.05a | 1.52±0.06a | 1894±35b |
|  | 10-20 cm | 379.42±5.10 | 4.28±0.09b | 0.90±0.15a | 0.96±0.02a | 0.70±0.07a | 1.54±0.03a | 1599±54b |
| HZ | 20-30 cm | 273.48±4.35 | 3.83±0.15b | 1.10±0.06b | 0.90±0.01a | 0.75±0.02a | 1.80±0.01a | 1323±30b |
|  | 30-40 cm | 190.52±2.62 | 3.13±0.09b | 1.14±0.05c | 0.86±0.02a | 0.86±0.10a | 1.80±0.04a | 1299±52a |
|  | 40-50 cm | 172.63±5.37 | 3.04±0.06ab | 2.70±0.18b | 0.89±0.02a | 0.98±0.11a | 1.84±0.05a | 688±16a |
|  | 0-10 cm | 610.33±6.79 | 6.55±0.11a | 0.57±0.02b | 0.99±0.01a | 0.60±0.04a | 1.46±0.07a | 2320±32a |
|  | 10-20 cm | 453.33±7.96 | 6.08±0.13a | 0.68±0.015a | 0.97±0.01a | 0.72±0.07a | 1.53±0.06a | 2074±45a |
| JGDQ | 20-30 cm | 294.77±5.52 | 5.47±0.12a | 1.59±0.070a | 0.95±0.01a | 0.83±0.06a | 1.67±0.09a | 1522±32a |
|  | 30-40 cm | 211.43±7.05 | 3.67±0.09a | 2.03±0.13a | 0.85±0.03a | 0.90±0.12a | 1.92±0.12a | 869±33c |
|  | 40-50 cm | 195.85±5.84 | 2.87±0.09b | 3.49±0.12a | 0.79±0.07a | 1.07±0.11a | 2.02±0.10a | 454±23b |

**Table S2.** Percentages of five fluorescent components in water sanmples at soil depths of 0-10cm, 10-20cm, 20-30cm, 30-40cm and 40-50cm at the continuous permafrost zone (TQ), discontinuous permafrost zone (HZ), isolated patches permafrost zone (JGDQ) (Mean ± SD).

| Location | Soil depth | C1 | C2 | C3 | C4 | C5 |
| --- | --- | --- | --- | --- | --- | --- |
|  | 0-10 cm | 31.07%±4.2% | 25.32%±3.2% | 30.17%±4.2% | 9.59%±2.1% | 3.85%±0.6% |
|  | 10-20 cm | 25.06%±2.8% | 23.58%±2.9% | 29.42%±3.3% | 6.86%±1.0% | 15.08%±1.1% |
| TQ | 20-30 cm | 25.34%±1.6% | 27.75%±1.5% | 9.71%±1.8% | 12.37%±1.6% | 25.24%±3.7% |
|  | 30-40 cm | 25.51%±2.9% | 23.00%±4.0% | 11.61%±1.6% | 11.82%±0.9% | 26.06%±2.1% |
|  | 40-50 cm | 29.74%±3.1% | 18.66%±0.6% | 4.05%±0.5% | 10.50%±1.3% | 37.14%±3.6% |
|  | 0-10 cm | 34.55%±0.9% | 26.51%±0.8% | 23.55%±0.3% | 12.52%±0.3% | 3.10%±0.6% |
|  | 10-20 cm | 26.65%±2.0% | 25.77%±0.8% | 28.01%±0.2% | 9.22%±2.0% | 10.36%±1.1% |
| HZ | 20-30 cm | 25.46%±1.9% | 29.92%±1.5% | 10.68%±2.8% | 11.81%±0.5% | 21.52%±0.8% |
|  | 30-40 cm | 23.16%±1.1% | 24.08%±0.7% | 21.56%±1.3% | 19.28%±0.9% | 22.02%±1.9% |
|  | 40-50 cm | 23.57%±2.1% | 16.04%±0.6% | 5.38%±0.2% | 15.01%±1.0% | 40.02%±3.2% |
|  | 0-10 cm | 41.50%±2.1% | 27.63%±1.5% | 13.32%±0.8% | 16.22%±3.6% | 1.35%±0.2% |
|  | 10-20 cm | 30.91%±1.7% | 30.29%±1.0% | 27.21%±1.2% | 9.15%±1.3% | 2.44%±0.7% |
| JGDQ | 20-30 cm | 25.86%±1.6% | 25.94%±1.6% | 31.71%±1.8% | 7.84%±1.1% | 8.65%±0.6% |
|  | 30-40 cm | 22.16%±0.9% | 22.56%±1.1% | 17.81%±1.0% | 7.36%±0.8% | 30.21%±4.3% |
|  | 40-50 cm | 15.09%±1.2% | 14.27%±3.2% | 9.37%±0.9% | 5.47%±0.8% | 55.08%±2.2% |

**Fig. S1.** Variations of BIX (a), FI(b), HIX(c) in soil under different permafrost zones (continuous permafrost, discontinuous permafrost, isolated patches permafrost) (*p* < 0.05).

**Fig. S2.** Pearson correlation analysis between different dissolved organic matter parameters of different permafrost zones (continuous permafrost, discontinuous permafrost, isolated patches permafrost). The circles' dimensions reflect the magnitude of the correlation coefficient, with deeper colors signifying a stronger correlation. A red circle represents a positive correlation, while a blue one indicates a negative correlation; the varying colors correspond to different correlation coefficient values.

^⁎^ Indicates significant correlation at the 0.05 level.

^⁎⁎^ Indicates significant correlation at the 0.01 level.

^⁎⁎⁎^ Indicates significant correlation at the 0.001 level.

**Fig. S3.** The ^1^H-NMR spectral characteristics of DOM at soil depths of 0-10, 10-20, 20-30, 30-40 and 40-50 cm (a: TQ, b: HZ, c JGDQ). The relative abundances of MDLT, CRAM, carbohydrates and arom (d: TQ, e: HZ, f: JGDQ).

**Solution-State ^1^H-NMR Test**

Weigh 20 mg of DOM powder and dissolve it in 1100 µL NaO/D_2_O solution (pH = 14) using ultrasound. After centrifugation, the liquid solution is transferred to a Bruker Bio Spin 5 mm NMR tube. Samples were analyzed by a BioSpin Avance III 400 MHz NMR spectrometer (Bruker Company, Karlsruhe, Germany). The loop delay time and time domain points were set to 2 s and 32 K, respectively, while 256 scans were acquired. Spectra were further processed with a zero-fflling factor of 2 and were apodised by multiplication with an exponential decay corresponding to 2.0 Hz line broadening. The integration of four regions was conducted based on the one-dimensional ^1^H-NMR spectra. These regions comprised (1) MDLT, referring to materials derived from linear terpenoids, with a chemical shift range of 0.6–1.6 ppm; (2) CRAM, representing carboxyl-rich alicyclic molecules, within the range of 1.6–3.2 ppm; (3) carb, indicating carbohydrates and peptides, spanning from 3.2 to 4.5 ppm; and (4) arom, signifying aromatics and phenolics, with a chemical shift range of 6.5–8.4 ppm.

**Table S3.** ^1^H-NMR of soil DOM in the 0-50 cm layer in different types of permafrost zones.

| Location | Soil depth | arom | carb | CRAM | MDLT |
| --- | --- | --- | --- | --- | --- |
|  | 0-10 cm | 11.60 | 23.66 | 29.43 | 35.31 |
|  | 10-20 cm | 9.48 | 21.05 | 27.98 | 41.48 |
| TQ | 20-30 cm | 8.63 | 19.91 | 25.03 | 46.43 |
|  | 30-40 cm | 9.65 | 17.72 | 23.82 | 48.81 |
|  | 40-50 cm | 8.97 | 16.61 | 25.58 | 48.84 |
|  | 0-10 cm | 11.61 | 24.71 | 31.16 | 32.53 |
|  | 10-20 cm | 7.19 | 23.76 | 32.98 | 36.06 |
| HZ | 20-30 cm | 7.00 | 23.93 | 31.68 | 37.38 |
|  | 30-40 cm | 5.34 | 26.84 | 35.05 | 32.77 |
|  | 40-50 cm | 3.93 | 28.75 | 34.62 | 32.69 |
|  | 0-10 cm | 13.32 | 28.85 | 30.27 | 27.55 |
|  | 10-20 cm | 7.94 | 26.32 | 32.45 | 33.28 |
| JGDQ | 20-30 cm | 5.30 | 31.29 | 32.17 | 31.22 |
|  | 30-40 cm | 4.02 | 29.68 | 38.44 | 27.84 |
|  | 40-50 cm | 3.45 | 33.72 | 36.19 | 26.62 |
